# Supplementary material for: Protein phosphatase 1 regulates atypical mitotic and meiotic division in Plasmodium sexual stages
Source: Commun Biol. 2021 Jun 18;4:760. doi: 10.1038/s42003-021-02273-0 (PMC8213788; doi:10.1038/s42003-021-02273-0)
Supplement: Supplementary file 2 — Supplementary Information [file 42003_2021_2273_MOESM2_ESM.pdf]

Supplementary information

Fig. S1. Generation and genotypic analysis of PP1GFP parasites

(a) Schematic representation for 3'-tagging of *pp1* gene with green fluorescent protein (GFP) sequence via single homologous recombination. **(b)** Integration PCR showing correct integration of tagging construct. **(c)** Western blot showing expected size of PP1-GFP protein. Markers sizes are given to the left of the panel.

Fig. S2. Generation and genotype analysis of *conditional knockdown PP1* parasites

(a) Schematic representation of auxin inducible degron (AID) strategy to generate PP1AID parasites. **(b)** Integration PCR of the PP1AID construct in the *pp1* locus. Primer 1 and Primer 2 were used for control PCR while primer 1 and primer 3 were used to determine successful integration of AID-HA sequence and selectable marker at 3'-end of *pp1* locus **(c)** PP1AID protein expression level as measured by western blotting upon addition of auxin to mature purified gametocytes; α-tubulin serves as a loading control. Auxin treatment of PP1AID showed no defect in exflagellation (error bars show standard deviation from the mean; technical replicates from three independent infections. **(d)** Schematic representation of the promoter swap strategy (PP1PTD, placing *pp1* under the control of the *ama1* promoter) by double homologous recombination. Arrows 1 and 2 indicate the primer positions used to confirm 5' integration and arrows 3 and 4 indicate the primers used for 3' integration. **(e)** Integration PCR of the promotor swap construct into the *pp1* locus. Primer 1 (5'-IntPTD36) with primer 2 (5'-IntPTD) were used to determine successful integration of the selectable marker. Primer 3 (3'-intPTama1) and primer 4 (3'-IntPTD36) were used to determine the successful integration of ama1 promoter. Primer 1 (5'-IntPTD36) and

primer 4 (3'-IntPTD36) were used to show complete knock-in of the construct and the absence of a band at 2.1 kb (endogenous) resulting in complete knock-in of the construct. **(f)** Parasitaemia during blood stage schizogony showing a significant slow growth of PP1PTD compared to WTGFP parasites. Experiment was done with three mice each with 1000 parasites per mice injected intraperitonially. ***P<0.001 **(g)** Bite back experiments show no transmission of PP1PTD parasites (black bar) from mosquito to mouse, while successful transmission was shown by WT-GFP parasites. Mean ± SD; n= 3 independent experiments.

Fig. S3. Analysis of PP1PTD development and RNA seq analysis:

(a) The quantification of electron microscopy data showing PP1PTD male gametocytes halted at an early stage of development in comparison with WTGFP male gametocytes at 30 min post activation. These data are based on analysis of fifty random sections of male gametocytes per sample.

(b) Clustered dendrogram of two biological replicates of WTGFP and PP1PTD mutant parasite lines during gametocyte stage using hierarchical clustering algorithm. Analysis was performed on normalized count data**. (c)** RNA-seq read statistics.

Fig S1

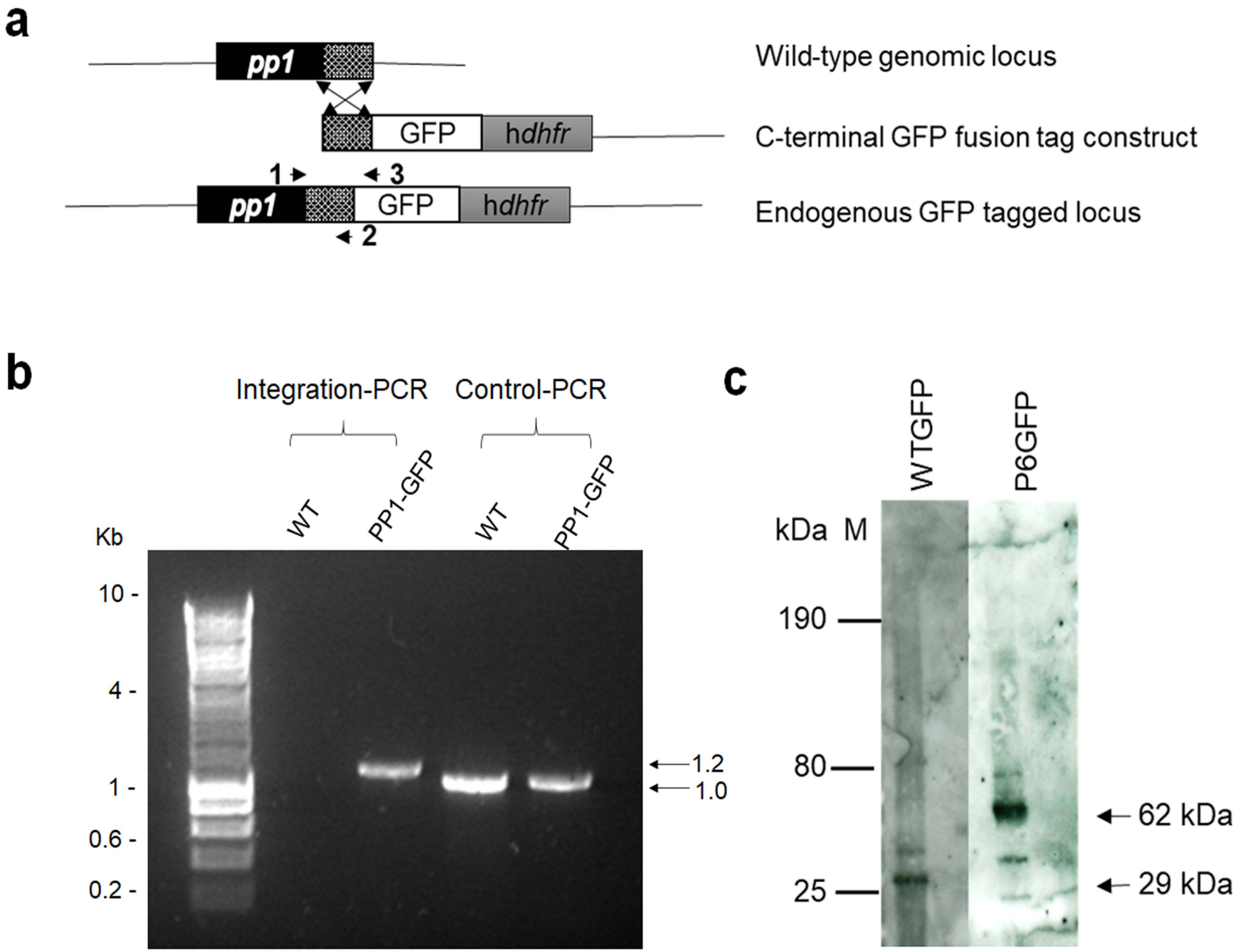
a
pp1
GFP
hdhfr
1
2
3
Wild-type genomic locus
C-terminal GFP fusion tag construct
Endogenous GFP tagged locus
b
Integration-PCR
Control-PCR
WT
PP1-GFP
WT
PP1-GFP
Kb
10 -
4 -
1 -
0.6 -
0.2 -
1.2
1.0
c
WTGFP
P6GFP
kDa M
190
80
25
62 kDa
29 kDa

Fig S2

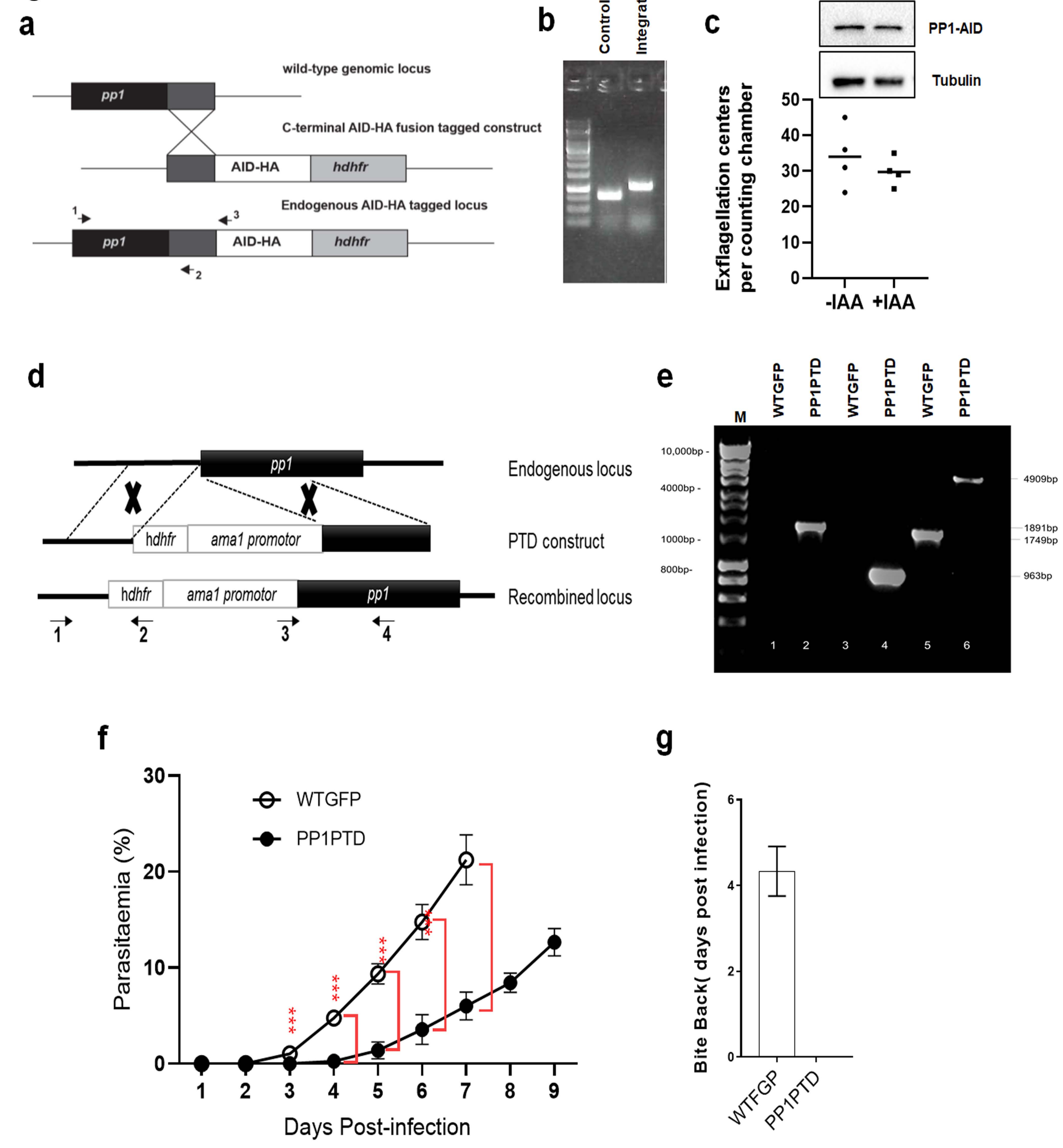

a
wild-type genomic locus
pp1
C-terminal AID-HA fusion tagged construct
AID-HA
hdhfr
Endogenous AID-HA tagged locus
1
2
3
b
Control
Integration
c
IAA
–
+
PP1-AID
Tubulin
Exflagellation centers per counting chamber
0
10
20
30
40
50
-IAA
+IAA
d
Endogenous locus
PTD construct
ama1 promotor
Recombined locus
4
e
M
WTGFP
PP1PTD
10,000bp -
4000bp -
1000bp -
800bp-
4909bp
1891bp
1749bp
963bp
5
6
f
WTGFP
PP1PTD
Parasitaemia (%)
Days Post-infection
***
7
8
9
g
Bite Back(days post infection)
WTFGP

Fig S3

a

Gametocytes (n=50)	Developmental stages (%) at 30 min post activation	
	**Early stages** (showing basal bodies, nuclear poles, axonemes)	**Late stages** (showing chromosome condensation, exflagellation)
WTGFP	17	83
PP1PTD	85	15

b

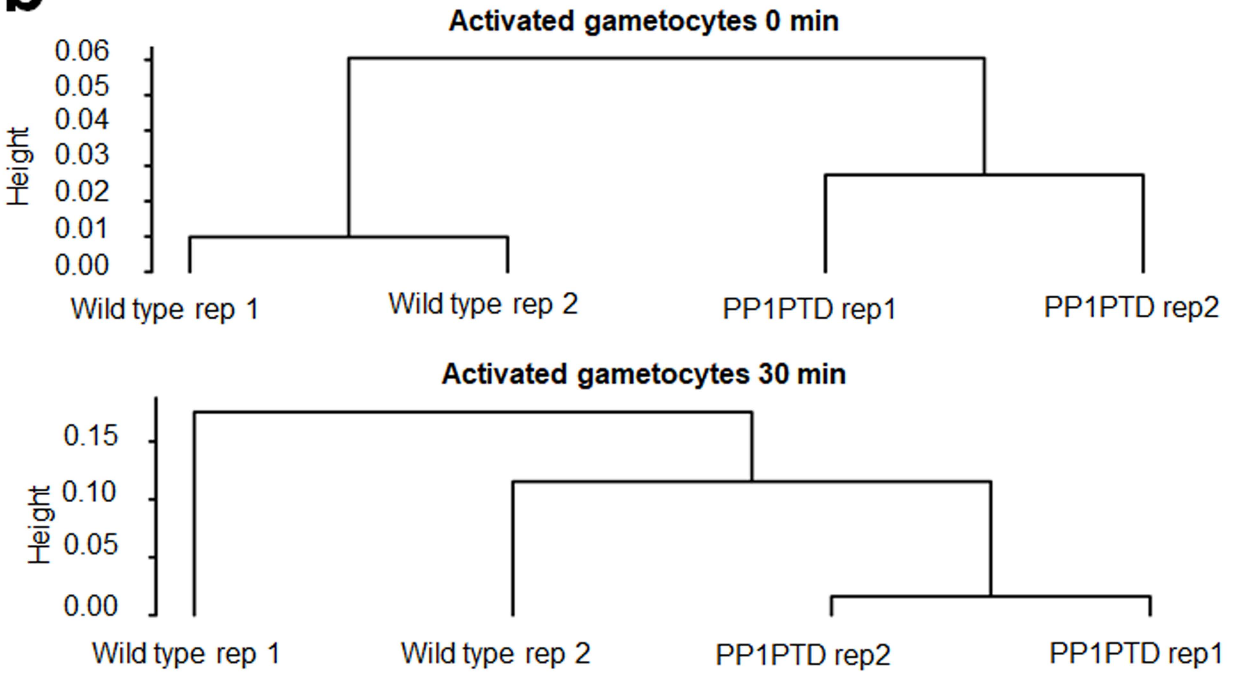

c

	**Wild type (in millions)**		**PP1PTD (in millions)**	
**Gametocytes**	Replicate 1	Replicate 2	Replicate 1	Replicate 2
**0 min**	45.99	25.5	58.6	19.8
**30 min**	22.57	31.57	16.33	20.65
