# Supplementary material for: Protein phosphatase 1 regulates atypical mitotic and meiotic division in Plasmodium sexual stages
Source: Commun Biol. 2021 Jun 18;4:760. doi: 10.1038/s42003-021-02273-0 (PMC8213788; doi:10.1038/s42003-021-02273-0)
Supplement: Supplementary file 3 — Description of Supplementary Files [file 42003_2021_2273_MOESM3_ESM.pdf]

Description of Additional Supplementary Files

File name: Supplementary Data 1

Description: Raw data related to phenotypic analysis.

File name: Supplementary Data 2

Description: Differentially expressed genes in PP1PTD parasites.

File name: Supplementary Data 3

Description: List of proteins pulled down by PP1GFP.

File name: Supplementary Data 4

Description: Primers used in this study.
