# Supplementary material for: Protein phosphatase 1 regulates atypical mitotic and meiotic division in Plasmodium sexual stages
Source: Commun Biol. 2021 Jun 18;4:760. doi: 10.1038/s42003-021-02273-0 (PMC8213788; doi:10.1038/s42003-021-02273-0)
Supplement: Supplementary file 8 — Reporting Summary [file 42003_2021_2273_MOESM8_ESM.pdf]

nature research

Corresponding author(s): DBPR-COMMSBIO-21-0254-T

Last updated by author(s): May 20, 2021

Reporting Summary

Nature Research wishes to improve the reproducibility of the work that we publish. This form provides structure for consistency and transparency in reporting. For further information on Nature Research policies, see our Editorial Policies and the Editorial Policy Checklist.

Statistics

For all statistical analyses, confirm that the following items are present in the figure legend, table legend, main text, or Methods section.

n/a	Confirmed	
☐	☒	The exact sample size (*n*) for each experimental group/condition, given as a discrete number and unit of measurement
☐	☒	A statement on whether measurements were taken from distinct samples or whether the same sample was measured repeatedly
☐	☒	The statistical test(s) used AND whether they are one- or two-sided<br>*Only common tests should be described solely by name; describe more complex techniques in the Methods section.*
☒	☐	A description of all covariates tested
☒	☐	A description of any assumptions or corrections, such as tests of normality and adjustment for multiple comparisons
☐	☒	A full description of the statistical parameters including central tendency (e.g. means) or other basic estimates (e.g. regression coefficient) AND variation (e.g. standard deviation) or associated estimates of uncertainty (e.g. confidence intervals)
☒	☐	For null hypothesis testing, the test statistic (e.g. *F*, *t*, *r*) with confidence intervals, effect sizes, degrees of freedom and *P* value noted<br>*Give P values as exact values whenever suitable.*
☒	☐	For Bayesian analysis, information on the choice of priors and Markov chain Monte Carlo settings
☒	☐	For hierarchical and complex designs, identification of the appropriate level for tests and full reporting of outcomes
☒	☐	Estimates of effect sizes (e.g. Cohen's *d*, Pearson's *r*), indicating how they were calculated

Our web collection on statistics for biologists contains articles on many of the points above.

Software and code

Policy information about availability of computer code

Data collection	NA
Data analysis	NA

For manuscripts utilizing custom algorithms or software that are central to the research but not yet described in published literature, software must be made available to editors and reviewers. We strongly encourage code deposition in a community repository (e.g. GitHub). See the Nature Research guidelines for submitting code & software for further information.

Data

Policy information about availability of data

All manuscripts must include a data availability statement. This statement should provide the following information, where applicable:

- Accession codes, unique identifiers, or web links for publicly available datasets
- A list of figures that have associated raw data
- A description of any restrictions on data availability

-Accession codes, unique identifiers, or web links for publicly available datasets

RNA Sequence reads have been deposited in the NCBI 629 Sequence gene expression omnibus with the accession number GSE164175. 630 "The mass spectrometry proteomics data have been deposited to the 631 ProteomeXchange Consortium with the dataset identifier PXD023571 and 632 10.6019/PXD023571.

-A list of figures that have associated raw data
Fig 4-raw data for phenotypic analysis (Supplementary data 1)

Fig 6- RNA seq data (Supplementary data 2)
Fig 7- Mass spectrometry data(Supplementary data 3)
Material and methods- Primers used in the study (Supplementary data 4)

- A description of any restrictions on data availability
None

Field-specific reporting

Please select the one below that is the best fit for your research. If you are not sure, read the appropriate sections before making your selection.

☒ Life sciences ☐ Behavioural & social sciences ☐ Ecological, evolutionary & environmental sciences

For a reference copy of the document with all sections, see nature.com/documents/nr-reporting-summary-flat.pdf

Life sciences study design

All studies must disclose on these points even when the disclosure is negative.

Sample size	Sample size for mice use were calculated using power calculation on EDA as given on NC3R (https://eda.nc3rs.org.uk/experimental-design-group#poweranalysis) considering certain values like significance level ≤0.05, when in reality there is a biologically relevant effect as a power of 0.9, an estimated variability of 0.5 in terms of standard deviation and a minimum difference of 2 between experiment and control that was analysed by 2-side test. All statistical analyses were performed using GraphPad Prism 8 (GraphPad Software) and an unpaired t-test and two way anova test were used to examine significant differences between wild-type and mutant strains for qRT PCR and phenotypic analysis accordingly.
Data exclusions	All the data is provided in supplementary files
Replication	We repeated experiments two to three times with both biological and technical replicates to establish any variation in result and rigorous reproducibility. ll statistical analyses were performed using GraphPad Prism 8 (GraphPad Software) and an unpaired t-test and two way anova test were used to examine significant differences between wild-type and mutant strains for qRT PCR and phenotypic analysis accordingly.
Randomization	We used female mice of age group ranging from 6 to 8 weeks randomly throughout the project duration. The parasite infection were initiated by all the license holders involved in this study in different batches of mice belonging to two different outbred strains (CD1 and TO) from two different suppliers (Charles River and Harlan).
Blinding	Phenotyping and data scoring and analysis were performed with the researcher blind to the genotype of the parasite. For proteomics experiments, it is not possible to blind the different tag lines used while growing in the mice and purification. However, the mass spec was done by the staff at University of Warwick who will not know the phenotype of the parasite. EM and expansion microscopy were performed blind by the staff at Oxford who were not aware of the phenotype of the samples. RNA sequencing analysis was performed at KAUST, Saudi Arabia blindly without any prior information about the samples.

Reporting for specific materials, systems and methods

We require information from authors about some types of materials, experimental systems and methods used in many studies. Here, indicate whether each material, system or method listed is relevant to your study. If you are not sure if a list item applies to your research, read the appropriate section before selecting a response.

Materials & experimental systems

n/a	Involved in the study	
☒	☐	Antibodies
☒	☐	Eukaryotic cell lines
☒	☐	Palaeontology and archaeology
☐	☒	Animals and other organisms
☒	☐	Human research participants
☒	☐	Clinical data
☒	☐	Dual use research of concern

Methods

n/a	Involved in the study	
☒	☐	ChIP-seq
☒	☐	Flow cytometry
☒	☐	MRI-based neuroimaging

Animals and other organisms

Policy information about studies involving animals; ARRIVE guidelines recommended for reporting animal research

Laboratory animals	Mice, outbred, TO/CD1 strain, female, 6-10 weeks.
Wild animals	*Provide details on animals observed in or captured in the field; report species, sex and age where possible. Describe how animals were caught and transported and what happened to captive animals after the study (if killed, explain why and describe method; if released, say where and when) OR state that the study did not involve wild animals.*
Field-collected samples	*For laboratory work with field-collected samples, describe all relevant parameters such as housing, maintenance, temperature, photoperiod and end-of-experiment protocol OR state that the study did not involve samples collected from the field.*
Ethics oversight	The animal work performed in the UK passed an ethical review process and was 461 approved by the United Kingdom Home Office. Work was carried out under UK 462 Home Office Project Licenses (30/3248 and PDD2D5182) in accordance with the 463 United Kingdom 'Animals (Scientific Procedures) Act 1986'.

Note that full information on the approval of the study protocol must also be provided in the manuscript.
